# Psychosocial Risk and Health Behaviors as Predictors of Clinical Events in Patients Wait-Listed for a New Heart: Results from 7 Years of Follow-Up

**DOI:** 10.3390/life11121438

**Published:** 2021-12-20

**Authors:** Kathleen Gali, Gerdi Weidner, Jacqueline M. A. Smits, Jan Beyersmann, Heike Spaderna

**Affiliations:** 1 Hamburg Center for Health Economics, University of Hamburg, 20354 Hamburg, Germany; kathleen.gali@uni-hamburg.de; 2 University Cancer Center Hamburg (UCCH), University Medical Center-Eppendorf (UKE), 20251 Hamburg, Germany; 3 Department of Biology, Romberg Tiburon Campus, San Francisco State University, Tiburon, CA 94920, USA; 4 Eurotransplant International Foundation, 2301 Leiden, The Netherlands; jsmits@eurotransplant.org; 5 Institute of Statistics, Ulm University, 89081 Ulm, Germany; jan.beyersmann@uni-ulm.de; 6 Department of Nursing Science, Section Health Psychology, Trier University, 54286 Trier, Germany

**Keywords:** advanced heart failure, heart transplant, depression, smoking, physical activity, dietary habits

## Abstract

We examined the long-term relationship of psychosocial risk and health behaviors on clinical events in patients awaiting heart transplantation (HTx). Psychosocial characteristics (e.g., depression), health behaviors (e.g., dietary habits, smoking), medical factors (e.g., creatinine), and demographics (e.g., age, sex) were collected at the time of listing in 318 patients (82% male, mean age = 53 years) enrolled in the Waiting for a New Heart Study. Clinical events were death/delisting due to deterioration, high-urgency status transplantation (HU-HTx), elective transplantation, and delisting due to clinical improvement. Within 7 years of follow-up, 92 patients died or were delisted due to deterioration, 121 received HU-HTx, 43 received elective transplantation, and 39 were delisted due to improvement. Adjusting for demographic and medical characteristics, the results indicated that frequent consumption of healthy foods (i.e., foods high in unsaturated fats) and being physically active increased the likelihood of delisting due improvement, while smoking and depressive symptoms were related to death/delisting due to clinical deterioration while awaiting HTx. In conclusion, psychosocial and behavioral characteristics are clearly associated with clinical outcomes in this population. Interventions that target psychosocial risk, smoking, dietary habits, and physical activity may be beneficial for patients with advanced heart failure waiting for a cardiac transplant.

## 1. Introduction

For patients with advanced heart failure, the wait for a heart transplantation (HTx) can be long. According to Eurotransplant, in 2020 there were approximately 350 heart transplants and 700 people in Germany on the heart waiting list [1,2]. The waiting period can be difficult for patients, both physically and psychologically [3,4]. Due to illness severity, some patients are admitted to the hospital and monitored to ensure satisfactory condition until transplantation [3]. The most common clinical outcomes for patients on the waiting list are death, heart transplantation (either high-urgency or elective), and delisting due to either clinical deterioration or improvement.

Psychosocial and lifestyle factors have been shown to play a role in outcomes in patients with heart failure. For example, depression and social isolation increase hospitalization and mortality [5,6,7]. Also detrimental to patients with heart failure is having a history of smoking. For example, active cigarette smoking, as well as heavy former smoking, increase the risk of heart failure, and risk of rehospitalizations and death in cardiac patients compared to those who have never smoked [8,9,10,11]. In contrast, engaging in healthy lifestyle behaviors can decrease the risk of having a poor outcome. For example, in older adults with advanced chronic heart failure, moderate physical activity was found to decrease the risk of death [12], and healthy dietary habits, such as eating a diet low in saturated fats and high in polyunsaturated fatty acids, have been shown to be associated with lower all-cause mortality [13]. Whether these factors affect clinical outcomes over the long term while waiting for a new heart is currently unclear.

To evaluate the role of psychosocial and behavioral factors in this population, the Waiting for a New Heart Study was conducted. The Waiting for a New Heart Study [14] is a prospective multi-site observational study of 318 patients newly registered for a heart transplant. The study evaluates the contributions of psychosocial risk (i.e., depression, social isolation) and behavioral factors (i.e., dietary habits, physical activity, and smoking) at the time of listing on clinical outcomes (i.e., death/delisting due to deterioration, high-urgency heart transplantation, elective transplantation, and delisting due to improvement). In previous reports using data from the Waiting for a New heart Study, based on shorter follow-ups (i.e., 1 year) and therefore fewer events, individual predictors were analyzed and outcomes such as event-free survival were considered. Low depressive symptoms, low psychosocial risk (defined as being socially integrated and not having depression), high physical activity, and healthy dietary habits (i.e., a diet high in poly- and mono-unsaturated fatty acids) were found to independently decrease the risk of adverse events, while having low social support (specifically in men) and smoking increased the risk of death [15,16,17,18,19].

This extended follow-up report of 7 years employed competing risks analysis to more accurately estimate the probability of an event of interest in the presence of competing outcome events compared to traditional methods. Specifically, we examined depressive symptoms, psychosocial risk, social support, dietary habits, physical activity, and smoking habits with the Waiting for a New Heart data consisting of over 7 years of follow-up.

With a longer follow-up we seek to understand the joint contribution of these factors on four major clinical events (death/delisting due to deterioration, high-urgency heart transplantation, elective transplantation, and delisting due to improvement). This comprehensive analytical approach allows for the examination of multiple risk factors that usually co-occur and can facilitate the development of prevention programs that target modifiable behavioral risk factors.

## 2. Materials and Methods

### 2.1. Study Population

The present analyses focused on baseline data from the Waiting for a New Heart Study obtained at the time of listing and on clinical outcomes collected during the waiting period until the last follow-up in February 2013. An invitation letter was sent to all heart transplant clinics in Germany and Austria by Eurotransplant. Sixteen hospitals in Germany and one in Austria agreed to participate. Comparisons of nonparticipants with participating patients have been reported previously [20]. The study was approved by local ethics committees and carried out in accordance with the Declaration of Helsinki.

### 2.2. Procedures and Participants

**Figure 1:**
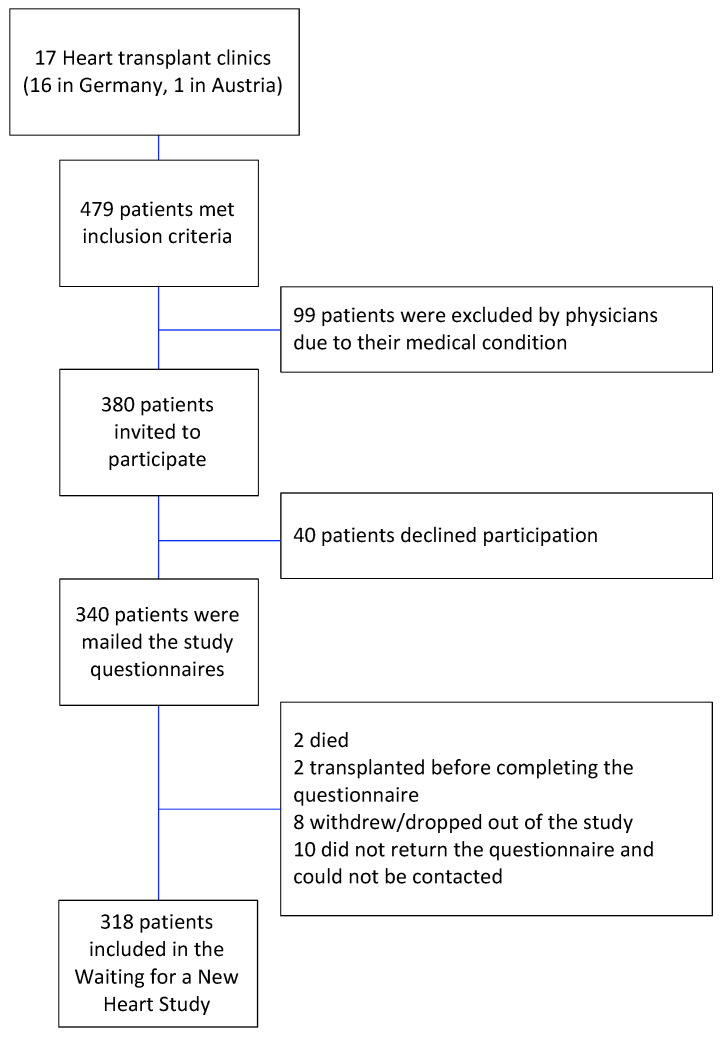
Flowchart illustrating participant recruitment.

The study procedures have been described in detail previously [20]. Shortly, informed consent was obtained from patients who were newly registered on the waiting list between April 2005 and December 2006. Patients were eligible for inclusion in the study if they were registered on the Eurotransplant HTx waiting list, 18 years or older at the time of listing, able to speak German fluently, had not received a donor heart before, and did not require a combined heart–lung transplantation. There were 380 patients in 17 hospitals who met the inclusion criteria and were invited to participate. Of these, 340 patients consented and were sent the questionnaires. Complete responses were obtained from 318 patients, yielding a 93.5% response rate (Figure 1).

### 2.3. Variables

#### 2.3.1. Waiting List Outcome Variables

Outcomes were based on data of patients’ changes in waiting list status as transferred by Eurotransplant. Outcome variables were: (1) death and delisting due to deterioration, (2) high-urgency status heart transplantation (HU-HTx), (3) elective transplantation (elective HTx), and (4) delisting due to clinical improvement. As delisting due to deterioration had the lowest cumulative incidence, it was combined with death for a combined outcome variable. High-urgency status, at the time of listing, was a temporary status applied to patients in intensive care units with cardiac index <2.2 L/m^2^/min and mixed venous oxygen saturation <55% while on inotropic therapy for at least 48 hours and beginning secondary organ failure [21]. High-urgency status was approved by Eurotransplant. Elective transplantation is transplantation while not in high-urgency status. Transplantation is influenced by the availability of a donor heart and match. Waiting list outcomes were based on type and date of waiting list status change until February 2013 since the date of wait-listing as provided by Eurotransplant. Patients who were still on the waiting list by the end of the follow-up, lost to follow- up, and delisted for other reasons (i.e., withdrawing consent for transplantation and delisted owing to noncompliance) were censored.

#### 2.3.2. Independent Variables

##### Psychosocial Risk Factors

Depressive symptoms were measured by the German version of the Hospital Anxiety and Depression Scale (HADS-D), which consisted of seven items (e.g., for depression: ‘‘I look forward with enjoyment to things’’ (reverse scored)) with scores ≥ 9 suggesting clinical depression [22]. Cronbach’s α in a German sample of 6200 patients (90% cardiology) was 0.81 for the depression scale [22] and α = 0.77 in our sample [20]. The English and German versions of the questionnaire have been extensively validated [23]. For analyses, depressive symptoms were dichotomized into high depressive symptoms (scores ≥ 9) and low depressive symptoms (scores < 9). Because anxiety was unrelated to waiting-list outcomes [19], it was not evaluated here. The number of social networks in the past month were assessed and used as an indicator for social isolation. Social isolation was indicated by a low network size, 0–4 persons/month in this study [24]. Psychosocial risk was a combination of the depression and social network scores; high risk was defined by the presence of both depression and social isolation (having ≤ 9 social networks) and low risk by having neither.

##### Dietary Habits

Dietary habits were measured using an adapted version of the *Fragebogen zur Erfassung des Gesundheitsverhaltens* (FEG), Questionnaire for the Assessment of Health Behavior [25]. It assesses consumption frequencies of 33 food items (e.g., bread, fresh fruits, salty snacks, butter, and fish). Participants indicated how often they consumed the listed foods (range: 4 = daily to 1 = never). Food items were categorized a priori according to the content of salt, and saturated and polyunsaturated and monounsaturated fatty acids [26], to calculate the frequency sum scores of intake of salty foods and foods high in polyunsaturated and monounsaturated fats (PUFA + MUFA). Item values were summed and then each sum value was divided by the number of included items [27].

##### Physical Activity

The number of physical activities patients engaged in at the time of listing was assessed using a modified version of the Community Healthy Activities Model Program for Seniors (CHAMPS), a Physical Activity Questionnaire for Older Adults [28]. Our adaptation [29] included 15 activities of light to moderate intensity, that is, with metabolic equivalents of task values (MET = 1 MET is a metabolic rate at rest consuming 3.5 mL of oxygen per kilogram of body weight per minute) between 2.5 and 6 according to the compendium of physical activities [30]. Items covered light gardening, walking to do errands, riding a bicycle, walking leisurely, Nordic walking, stretching, or light exercises. Participants were asked whether they regularly engaged in each activity over the past 4 weeks (yes/no). If yes, the weekly frequency for the specified activity was asked. To calculate caloric expenditure, participants were also asked to specify the number of hours per week they usually spent in this activity on a six-point scale from “less than one hour” to “9 or more hours”. The duration of time spent on an activity was weighted by its MET value and multiplied by 3.5 × 60 × (weight in kg/200). These caloric expenditure (kcal per week) values were then summed across all activities [19,28].

##### Smoking Status

Smoking status (current, former, and never) and year of quitting among former smokers were assessed by self-report [16]. Smoking status was grouped into three categories for analyses: current, former (quit ≤ 10 years ago), and non-smoking (never and quit > 10 years ago). Because never smokers and those who had quit more than 10 years have similar outcomes, they were grouped together for analyses [16].

##### Medical Variables

Medical variables at the time of listing were provided by Eurotransplant. These included anthropometric measurements, creatinine level, cardiac index, and the seven parameters to calculate the Heart Failure Survival Score (HFSS) [31]: mean arterial blood pressure, resting heart rate, left ventricular ejection fraction (LVEF), serum sodium, presence of intraventricular conduction delay (QRS interval ≥ 0.12 s), etiology of heart failure (ischemic versus non-ischemic), and peak oxygen consumption (peak VO_2_). Inpatient status at time of listing (yes/no) was assessed via questionnaire.

##### Demographic Characteristics

Demographic characteristics considered were age and gender.

### 2.4. Statistical Analyses

Analyses were conducted using R version 3.1.3 including the package *cmprsk*. A semiparametric multiple imputation procedure was used to handle missing medical parameters [29,32]. Analyses, except for competing risks regressions, were conducted across 10 imputed data sets and results pooled. Competing risks analyses were performed with the first of the ten imputed data sets and checked against the other nine records. To explore the robustness of the obtained results, analyses were repeated with non-imputed data.

Descriptive statistics are presented in absolute numbers and percentages for categorical variables and means and standard deviations for continuous variables. In addition, the minimum and maximum absolute numbers from categorical data and the minimum and maximum means and standard deviations observed in the imputed data are also presented.

**Figure 2:**
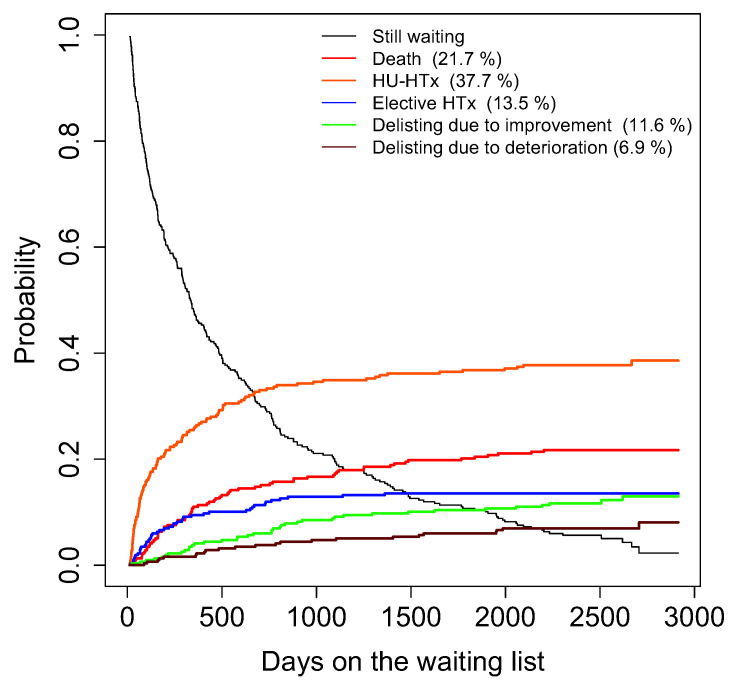
Cumulative incidence functions of each separate outcome in the Waiting for a New Heart Study.

We used competing risks regression models to identify psychological and behavioral predictors for each clinical outcome variable. Utilizing a competing risks approach, we considered the mutually exclusive outcomes death/delisting due to deterioration, high-urgency transplantation, elective transplantation, and delisting due to clinical improvement, whichever occurred first [33]. Therefore, all competing outcomes were considered in a competing risks regression simultaneously for which we report sub-distribution estimates for each outcome [34]. Sub-distribution hazard ratios (SHR) are interpreted similarly to a hazard ratio in a Cox regression in a qualitative manner. For instance, if the estimated sub-distribution hazard ratio for low psychosocial risk is greater than 1 when predicting delisting due to improvement, this suggests that low psychosocial risk is associated with higher incidence of delisting due to improvement while controlling for all covariates and the fact that death and heart transplantation can also occur while on the wait list. Cumulative incidence functions were plotted for each separate outcome, indicating the probability of the event of interest over the course of time (see Figure 2).

We first computed sub-distribution hazard ratios for each of the demographic, medical, psychological, and behavioral variables separately in univariate competing risks regression on all clinical outcomes. We then examined the effect of each risk factor (i.e., psychological and behavioral variables) on our clinical outcomes adjusting for standard covariates: age, sex, and heart failure severity (creatinine, cardiac index, HFSS, and inpatient status). We then took those factors associated with at least one of the clinical outcomes at *p* < 0.1 and included them in a comprehensive model that included relevant psychosocial and behavioral factors as well as our standard covariates. To avoid over-specification, variables highly correlated to one another were evaluated and were removed from the models. The proportional hazards assumption of included variables for the final multivariate model was evaluated by inspection of plotted scaled Schoenfeld residuals [35].

## 3. Results

### 3.1. Patient Characteristics

Psychosocial factors, health behaviors, medical characteristics, and demographics of 318 newly listed transplant candidates are presented in Table 1. The sample was mainly male (81.8%), with a mean age of 53.1 (SD = 11.1). Most (62.6%) had 9 years or less of education. Saturated fats and salt intake were highly correlated (*r* = 0.66) as were caloric expenditure and number of physical activities (*r* = 0.71). Therefore, saturated fats and caloric expenditure were not included in the competing risks analyses.

### 3.2. Waiting List Outcomes

Participants were observed for a mean follow-up of 623.8 days (SD = 712.3, median 335 days, range 12–2914 days). Cumulative incidence functions of outcomes are presented in Figure 1. Fourteen patients were still on the waiting list at the end of the follow-up and nine were delisted due to other reasons such as withdrawing consent for transplantation and being delisted owing to noncompliance.

### 3.3. Univariate Competing Risks Models

Table 2 and Table 3 report univariate associations between demographic factors, disease severity variables, psychological characteristics, and health behaviors on clinical outcomes. In univariate analyses, low depressive symptoms (SHR = 2.59, *p* = 0.02), low psychosocial risk (SHR = 2.58, *p* < 0.01), eating a diet high in PUFA + MUFA (SHR = 2.13, *p* < 0.05), and engaging in more physical activities (SHR = 1.17, *p* < 0.001) were associated with an increased rate of delisting due to improvement. A more frequent salty food intake was associated with a 2.6-fold likelihood for HU-HTx and subsequent reduced likelihood of being delisted due to death or deterioration (SHR = 0.56, *p* = 0.04). Low psychosocial risk also reduced the likelihood of being delisted due to death or deterioration (SHR = 0.29, *p* < 0.01). The number of physical activities (SHR = 0.88, *p* < 0.01) was significantly associated with a reduced likelihood of HU-HTx. On the other hand, social networks (SHR = 1.03, *p* < 0.01) increased the likelihood of HU-HTx. Eating a diet high in PUFA + MUFA (SHR = 0.52, *p* < 0.05) decreased the likelihood of elective HTx.

### 3.4. Multivariate Competing Risks Models

Psychosocial factors (depressive symptoms, psychosocial risk (includes depressive symptoms and social networks)), dietary habits (salty foods, PUFA + MUFA), physical activity, and smoking status were associated with at least one of the clinical outcomes at *p* < 0.1 in separate competing risks analyses adjusted for standard covariates (demographics and disease severity; Table 2). These relevant psychosocial and behavioral factors were then entered simultaneously, not separately as before, into a comprehensive competing risks model, except for depressive symptoms and inpatient status. Depressive symptoms were excluded because of its high correlation with psychosocial risks (which includes depressive symptoms and social networks) and inpatient status was excluded because of its moderate correlation with physical activity. Social networks and emotional support were not associated with any of the clinical outcomes after adjusting for demographics and disease severity and were therefore not considered further. After inspection, residuals did not show any indication of violation of proportionality except for physical activity in HU-HTx. An interaction term of physical activity and time was entered into the competing risks model for the HU-HTx outcome due to its time-varying effect. The reported SHR for physical activity on HU-HTx is the average effect across the waiting time.

In this multivariate model (Table 4), consuming more PUFA + MUFA (SHR = 2.27, *p* = 0.04) and engaging in more physical activity (SHR = 1.13, *p* = 0.03) remained significantly associated with being delisted due to improvement. However, the SHR of low psychosocial risk was slightly diminished and missed the level of statistical significance (SHR = 2.41, *p* = 0.052). While a higher number of physical activities engaged in at the time of listing increased the chances and was associated with a shorter time until delisting due to clinical improvement (SHR = 1.13, *p* = 0.03), it reduced the likelihood of receiving a HU-HTx (SHR = 0.79, *p* < 0.001). A high consumption of salty foods (SHR = 2.19, *p* = 0.01) increased the likelihood of HU-HTx, current smoking status increased the likelihood of delisting due to death or deterioration (SHR = 2.67, *p* = 0.03), and low psychosocial risk (SHR = 0.34, *p* = 0.02) decreased the likelihood of being delisted due to death or deterioration. Eating a diet high in PUFA + MUFA (SHR = 0.41, *p* = 0.04) remained associated with a decreased likelihood of receiving an elective HTx.

## 4. Discussion

In this long-term follow-up with over seven years of observation, adjusted competing risks models showed that having low psychosocial risk, eating healthy foods (high in PUFA + MUFA and low in salt), engaging in more physical activity, and not smoking improved clinical outcomes in heart failure patients awaiting a heart transplant.

Specifically, low psychosocial risk was found to decrease the likelihood of death/delisting due to deterioration. This finding is consistent with the 1 year follow-up in The Waiting for a New Heart Study, where psychosocial stress was found to contribute to event-free survival [36]. These findings, together with previous research pointing to an association of major depression with mortality in heart failure patients [37], highlight the importance of assessing psychosocial patient characteristics to facilitate the design of behavioral interventions. Unfortunately, depressive symptoms are often overlooked in clinical practice and can be difficult to diagnose in heart failure patients due to the overlapping symptoms with heart failure [38]. Nevertheless, addressing depression in heart failure patients has already showed promise. For example, in a clinical trial 158 patients with heart failure were randomized into a usual care group or a cognitive behavioral therapy (CBT) group that consisted of weekly 1 hour sessions for 6 months. Those in the CBT group had lower depressive symptoms at the 6 month follow- up than the usual care group [39].

Our findings on dietary habits are consistent with findings in heart health interventions. Patients with heart failure are commonly recommended to restrict dietary sodium, despite the limited evidence of the benefits [40,41]. However, adherence to low sodium restrictions (i.e., <2000 mg/d) is difficult for heart failure patients [42]. In this study, we found the consumption of foods high in salt increased the need for urgent heart transplantation in patients with rapidly deteriorating clinical status. In contrast, we also found consumption of foods high in PUFA + MUFA (healthy fats) decreased time to delisting due to improvement, which is in line with other studies that have shown a benefit to consuming foods with PUFA + MUFA [13,27,43,44]. Dietary guidelines should address various food groups as well as salt consumption. Salt consumption is correlated with higher fluid intake and consumption of saturated fatty acids [27], which can also lead to poor outcomes and worsening of symptoms [45,46]. To ensure a healthy diet rich in nutrients and that limits foods high in sodium, promotion of the DASH diet or a Mediterranean diet for heart failure patients could be helpful [47,48,49,50].

Research has shown the important role physical activity has played in the prognosis of heart failure. High levels of physical activity are associated with lower risks of incidence of heart failure [51], while inactivity in heart failure patients is associated with all-cause mortality and cardiac mortality [52]. Our study adds to the literature showing that physical activity can improve outcomes in patients listed for a heart transplant, specifically engagement in more physical activities can decrease the time to delisting due to improvement and is less likely to be associated with a high-urgent status heart transplantation. Therefore, encouragement of physical activity is important in clinical practice. However, barriers for patients with heart failure to engage in high levels of physical activity exist. One such barrier is the fear of physical activity (FoPA). Using the Fear of Activity in Situations-Heart Failure (FActS-HF) questionnaire [53], one study found fear, not anxiety or depression, was significantly associated with less physical activity in outpatients with heart failure [54].

Current smoking, independent of other risk factors, led to death and delisting due to deterioration. This finding is consistent with an earlier report of an association with smoking and death 1 year after being listed for a HTx in the Waiting for a New Heart Study [16]. This current study demonstrates that after a longer follow-up, the effect of smoking on severe adverse events in this population of heart transplant candidates was maintained. One review study found 16% of heart failure patients continue to smoke after diagnosis, and that persistent smoking not only leads to increased mortality, as we saw in our study, but is also associated with increased risk of readmissions, poor health status, ventricular tachycardia, and arterial stiffness [9]. It is also problematic that among those with a history of smoking who are able to receive a heart transplant have a high risk of poor outcomes post-heart transplantation [55]. One study found 26% of current and former smokers who all stopped prior to heart transplantation resumed smoking after transplantation [56]. Cigarette smoking is a known major risk factor for heart failure [8] and smoking cessation should be achieved as early as possible to decrease the risk of heart failure [57]. Interventions are needed to support heart failure patients who smoke to quit.

Including psychosocial patient characteristics in the evaluation before listing for HTx has been recommended in order to assess risk factors for poor outcomes and to collect information to characterize patients’ resources [58]. This includes health behaviors and substance use history. Our study highlights the fact that health behaviors such as dietary habits and physical activity, as well as psychosocial risk, are also important factors in this domain.

Among the study’s limitations are the reliance on self-reported behaviors and psychosocial variables. In addition, our results may not be generalizable to other transplant programs that may have different guidelines for listing. The strengths of this study include the utilization of a competing risks method which allowed us to identify the effect of various independent predictors while simultaneously considering multiple clinical outcomes that can occur in patients on the heart transplant waiting list. Other survival analyses, such as the Kaplan–Meier approach, only consider one endpoint and can thus lead to overestimation when competing outcomes are present. Examining multiple risk factors, compared to previous studies that looked only at a specific factor, we were able to take into account behavioral factors that often co-occur.

## 5. Conclusions

In conclusion, psychological characteristics and health behaviors were independently associated with four major clinical outcome categories investigated in this study population of heart transplant candidates, even after controlling for demographic characteristics and disease severity. However, psychosocial characteristics, dietary habits, physical activity, and smoking were each differentially related to outcomes. Specifically, physical activity and healthy eating habits were associated with increased chances of improvement and subsequently delisting due to improvement; high consumption of salty foods and physical inactivity were associated with high-urgency transplantation, a procedure necessary among patients with rapidly declining clinical status; and psychosocial risk and current smoking were related to severe adverse events (increased risk of death and deterioration). Understanding how these different factors contribute to prognosis in patients with advanced heart failure can be useful for the stabilization and management of patients waiting for a heart transplant.

## Figures and Tables

**Table 1:** Baseline characteristics of 318 patients newly listed for a heart transplantation.

		Original Data			Imputed Data (*n* = 318)			
		n	n/M	%/SD	M	%/SD	Min_M_/Min_n_	Max_M_/Max_n_
***Demographics***								
Gender, *n* (%)		318						
	Male		260	81.8%				
	Female		58	18.2%				
Age (years)		318	53.1	11.1				
Education, *n* (%)		318						
	≤9 Years		199	62.6%				
	>9 Years		119	37.4%				
***Medical Characteristics***								
HFSS ^a^		224	7.9	0.9	7.86	0.9	7.9	7.9
Creatinine		301	1.4	0.5	1.39	0.5	1.4	1.4
Cardiac Index		289	2.0	0.6	2.06	0.6	2.0	2.1
Inpatient		318						
	Yes		87	27.4%				
	No		231	72.6%				
***Body Mass Index***		318	25.9	4.0				
***Psychological Factors***								
Depressive symptoms, *n* (%)		318						
	Low		195	61.3%				
	High		123	38.7%				
Social network size		318	8.2	6.2				
Psychosocial risk ^b^, *n* (%)		318						
	Low		47	14.8%				
	High		271	85.2%				
Emotional support, *n* (%)		318						
	Low		58	18.2%				
	High		260	81.8%				
***Dietary Habits***								
Salt		318	2.1	0.4				
Saturated fatty acids		318	2.2	0.4				
PUFA + MUFA ^c^		318	2.3	0.4				
***Physical Activity***								
Number of Physical Activities		318	3.5	2.4				
Caloric expenditure (kcal/week)		318	17.0	17.2				
***Smoking status***		316						
	Non (never and quit > 10 y)		166	52.5%			166	168
	Former		138	43.7%			138	139
	Current		12	3.8%			12	13

Note: ^a^ HFSS = Heart Failure Survival Score; ^b^ psychosocial risk defined by depressive symptoms and being socially isolated; ^c^ PUFA + MUFA = polyunsaturated and monosaturated fats (healthy fats).

**Table 2:** Univariate outcome-specific sub-distribution hazard ratios (SHR) of competing waiting list outcomes associated with demographics and medical characteristics in 318 newly listed heart transplant candidates.

	Death/Deteriorated (*n* = 92)		HU-HTx (*n* = 121)		Elective HTx (*n* = 43)		Improved (*n* = 39)	
Independent Variable	SHR (95% CI)	*p*	SHR (95% CI)	*p*	SHR (95% CI)	*p*	SHR (95% CI)	*p*
*Demographics*								
Female	1.54 (0.95, 2.49)	0.08	0.79 (0.47, 1.33)	0.37	1.40 (0.69, 2.85)	0.35	0.65 (0.25, 1.67)	0.37
Age (years)	1.02 (1.00, 1.04)	0.07	0.99 (0.97, 1.00)	0.12	1.01 (0.98, 1.04)	0.51	0.99 (0.96, 1.02)	0.45
*Medical Characteristics*								
HFSS ^a^	0.81 (0.64, 1.02)	0.07	0.80 (0.66, 0.96)	0.02	1.06 (0.70, 1.60)	0.80	1.68 (1.32, 2.15)	<0.001
Cardiac Index	0.86 (0.64, 1.14)	0.29	0.52 (0.38, 0.72)	<0.001	1.36 (1.08, 1.73)	<0.01	1.65 (1.25, 2.18)	<0.001
Creatinine	1.45 (0.97, 2.17)	0.07	1.11 (0.79, 1.57)	0.55	1.01 (0.53, 1.91)	0.98	0.52 (0.23, 1.18)	0.12
Inpatient Status	0.44 (0.25, 0.79)	<0.01	3.22 (2.20, 4.71)	<0.001	1.54 (0.81, 2.89)	0.19	0.29 (0.10, 0.80)	0.02

Note: ^a^ HFSS = Heart Failure Survival Score.

**Table 3:** Univariate and adjusted outcome-specific sub-distribution hazard ratios (SHR) of competing waiting list outcomes associated with psychosocial factors, diet, physical activity, and substance use in 318 newly listed heart transplant candidates.

	Death/Deteriorated (*n* = 92)		HU-HTx (*n* = 121)		Elective HTx (*n* = 43)		Improved (*n* = 39)	
Independent Variable	SHR (95% CI)	*p*	SHR (95% CI)	*p*	SHR (95% CI)	*p*	SHR (95% CI)	*p*
*Psychosocial Factors*								
Low Depressive Symptoms _uni_	0.78 (0.52, 1.18)	0.23	0.80 (0.56, 1.15)	0.23	1.06 (0.57, 1.97)	0.85	2.59 (1.20, 5.60)	0.02
**Low Depressive Symptoms _adj_**	0.80 (0.53, 1.22)	0.30	0.81 (0.56, 1.17)	0.27	1.01 (0.55, 1.86)	0.98	2.52 (1.15, 5.51)	0.02
Social Networks _uni_	0.97 (0.94, 1.01)	0.11	1.03 (1.01, 1.06)	<0.01	0.98 (0.92, 1.04)	0.53	1.02 (0.98, 1.06)	0.42
**Social Networks _adj_**	0.98 (0.94, 1.01)	0.19	1.02 (0.99, 1.04)	0.28	0.98 (0.91, 1.05)	0.50	1.01 (0.96, 1.05)	0.77
Low Psychosocial Risk _uni_	0.29 (0.12, 0.73)	<0.01	1.53 (0.97, 2.43)	0.07	0.74 (0.29, 1.90)	0.54	2.58 (1.27, 5.26)	<0.01
**Low Psychosocial Risk _adj_**	0.31 (0.13, 0.78)	0.01	1.66 (1.02, 2.70)	0.04	0.67 (0.26, 1.72)	0.40	2.45 (1.07, 5.64)	0.04
Low Emotional Support _uni_	1.12 (0.67, 1.89)	0.66	0.83 (0.50, 1.36)	0.46	1.57 (0.80, 3.09)	0.19	0.81 (0.34, 1.95)	0.64
**Low Emotional Support _adj_**	1.24 (0.74, 2.08)	0.42	0.83 (0.49, 1.39)	0.48	1.69 (0.85, 3.37)	0.13	0.61 (0.23, 1.62)	0.32
*Dietary Habits*								
Salty Foods _uni_	0.56 (0.32, 0.98)	0.04	2.60 (1.45, 4.67)	<0.01	0.90 (0.36, 2.23)	0.81	0.67 (0.29, 1.53)	0.34
**Salty Foods _adj_**	0.58 (0.32, 1.06)	0.08	1.73 (0.93, 3.20)	0.08	1.21 (0.47, 3.11)	0.70	0.75 (0.28, 2.04)	0.57
PUFA + MUFA _uni_	0.76 (0.48, 1.19)	0.23	1.08 (0.71, 1.65)	0.72	0.52 (0.27, 0.99)	0.05	2.13 (1.01, 4.48)	0.05
**PUFA + MUFA _adj_**	0.74 (0.46, 1.19)	0.21	1.16 (0.76, 1.79)	0.49	0.47 (0.22, 0.98)	0.04	2.20 (1.03, 4.71)	0.04
*Physical Activity*								
Number of Physical Activities _uni_	0.97 (0.89, 1.05)	0.46	0.88 (0.81, 0.96)	<0.01	1.03 (0.91, 1.16)	0.68	1.17 (1.07, 1.27)	<0.001
**Number of Physical Activities _adj_**	1.00 (0.92, 1.08)	0.90	0.95 (0.87, 1.04)	0.28	1.05 (0.92, 1.19)	0.51	1.12 (1.01, 1.25)	0.03
*Smoking Status*								
Non-Smoking (Referent)								
Former _uni_	1.18 (0.78, 1.80)	0.44	0.88 (0.61, 1.26)	0.48	0.73 (0.39, 1.36)	0.32	0.92 (0.48, 1.75)	0.79
Current _uni_	2.38 (0.98, 5.79)	0.06	0.36 (0.09, 1.50)	0.16	0.50 (0.07, 3.47)	0.48	1.41 (0.32, 6.25)	0.65
**Former _adj_**	1.49 (0.95, 2.35)	0.09	0.74 (0.50, 1.10)	0.14	0.79 (0.41, 1.51)	0.47	0.77 (0.39, 1.49)	0.43
**Current _adj_**	2.49 (1.06, 5.85)	0.04	0.31 (0.07, 1.29)	0.11	0.56 (0.08, 3.92)	0.56	1.72 (0.39, 7.56)	0.47

Note: PUFA + MUFA = polyunsaturated fatty acids and monounsaturated fatty acids; uni = univariate models; adj = adjusted models (in bold) adjusted for age, sex, heart failure survival score, cardiac index, creatinine, and inpatient status.

**Table 4:** Final multivariate competing risks model of outcome-specific sub-distribution hazard ratios (SHR) of competing waiting list outcomes.

	Death/Deteriorated (*n* = 92)		HU-HTx (*n* = 121)		Elective HTx (*n* = 43)		Improved (*n* = 39)	
Independent Variable	SHR (95% CI)	*p*	SHR (95% CI)	*p*	SHR (95% CI)	*p*	SHR (95% CI)	*p*
*Psychosocial Factors*								
Low Psychosocial Risk	0.34 (0.13, 0.86)	0.02	1.45 (0.89, 2.37)	0.14	0.59 (0.23, 1.49)	0.26	2.41 (0.99, 5.83)	0.052
*Dietary Habits*								
Salty Foods	0.57 (0.31, 1.03)	0.06	2.19 (1.20, 3.99)	0.01	1.26 (0.49, 3.25)	0.63	0.61 (0.22, 1.71)	0.35
PUFA + MUFA	0.72 (0.44, 1.18)	0.20	1.18 (0.76, 1.84)	0.46	0.41 (0.18, 0.94)	0.04	2.27 (1.04, 4.96)	0.04
*Physical Activity*								
Number of Physical Activities	1.00 (0.92, 1.09)	0.95	0.79 (0.71, 0.89)	<0.001	1.06 (0.92, 1.23)	0.44	1.13 (1.02, 1.25)	0.03
*Smoking Status*								
Non-Smoking (Referent)								
Former	1.28 (0.83, 1.99)	0.26	0.78 (0.53, 1.16)	0.23	0.65 (0.30, 1.39)	0.27	0.87 (0.44, 1.73)	0.70
Current	2.67 (1.08, 6.61)	0.03	0.33 (0.08, 1.41)	0.13	0.48 (0.06, 3.67)	0.48	1.35 (0.21, 8.82)	0.75

Note: PUFA + MUFA = polyunsaturated fatty acids and monounsaturated fatty acids (healthy fats); all models adjusted for age, sex, heart failure survival score, cardiac index, creatinine, and the other behavioral factors.

## Data Availability

The data sets used and/or analyzed during the current study can be requested from the corresponding authors.

## References

[1] Eurotransplant Statistics Report Library (2021). Active Heart Waiting List (at Year End) in Eurotransplants, by Year, by Country.

[2] Eurotransplant Statistics Report Library (2021). Heart Transplants (Deceased Donor), by Year, by Country.

[3] Maltês S., Rocha B.M.L., Cunha G.J.L., Brízido C., Strong C., Tralhão A., Weigert A., Duarte J.S., Aguiar C., Mendes M. (2021). Challenges of Organ Shortage for Heart Transplant: Surviving Amidst the Chaos of Long Waiting Times. Transplant. Direct.

[4] Zipfel S., Löwe B., Paschke T., Immel B., Lange R., Zimmermann R., Herzog W., Bergmann G. (1998). Psychological distress in patients awaiting heart transplantation. J. Psychosom. Res..

[5] Rutledge T., Reis V.A., Linke S.E., Greenberg B.H., Mills P.J. (2006). Depression in heart failure a meta-analytic review of prevalence, intervention effects, and associations with clinical outcomes. J. Am. Coll. Cardiol..

[6] Kaiser P., Allen N., Delaney J.A.C., Hirsch C.H., Carnethon M., Arnold A.M., Odden M.C. (2020). The association of prediagnosis social support with survival after heart failure in the Cardiovascular Health Study. Ann. Epidemiol..

[7] Heidari Gorji M.A., Fatahian A., Farsavian A. (2019). The impact of perceived and objective social isolation on hospital readmission in patients with heart failure: A systematic review and meta-analysis of observational studies. Gen. Hosp. Psychiatry.

[8] Gopal D.M., Kalogeropoulos A.P., Georgiopoulou V.V., Smith A.L., Bauer D.C., Newman A.B., Kim L., Bibbins-Domingo K., Tindle H., Harris T.B. (2012). Cigarette smoking exposure and heart failure risk in older adults: The Health, Aging, and Body Composition Study. Am. Heart J..

[9] Son Y.-J., Lee H.-J. (2020). Association between persistent smoking after a diagnosis of heart failure and adverse health outcomes: A systematic review and meta-analysis. Tob. Induc. Dis..

[10] Ahmed A.A., Patel K., Nyaku M.A., Kheirbek R.E., Bittner V., Fonarow G.C., Filippatos G.S., Morgan C.J., Aban I.B., Mujib M. (2015). Risk of Heart Failure and Death After Prolonged Smoking Cessation. Circ. Heart Fail..

[11] Duncan M.S., Freiberg M.S., Greevy R.A., Kundu S., Vasan R.S., Tindle H.A. (2019). Association of Smoking Cessation With Subsequent Risk of Cardiovascular Disease. JAMA.

[12] Cacciatore F., Amarelli C., Ferrara N., Della Valle E., Curcio F., Liguori I., Bosco Q., Maiello C., Napoli C., Bonaduce D. (2020). Protective effect of physical activity on mortality in older adults with advanced chronic heart failure: A prospective observational study. Eur. J. Prev. Cardiol..

[13] Colin-Ramirez E., Castillo-Martinez L., Orea-Tejeda A., Zheng Y., Westerhout C.M., Ezekowitz J.A. (2014). Dietary fatty acids intake and mortality in patients with heart failure. Nutrition.

[14] Zahn D., Weidner G., Beyersmann J., Smits J.M., Deng M.C., Kaczmarek I., Meyer S., Reichenspurner H., Mehlhorn U., Wagner F.M. (2010). Composite risk scores and depression as predictors of competing waiting-list outcomes: The Waiting for a New Heart Study. Transpl. Int..

[15] Weidner G., Zahn D., Mendell N.R., Smits J.M., Deng M.C., Zittermann A., Spaderna H., Waiting for a New Heart Study Group (2011). Patients’ sex and emotional support as predictors of death and clinical deterioration in the waiting for a new heart study: Results from the 1-year follow-up. Prog Transpl..

[16] Gali K., Spaderna H., Smits J.M., Bramstedt K.A., Weidner G. (2016). Smoking Status at Time of Listing for a Heart Transplant Predicts Mortality on the Waiting List: A Multicenter Prospective Observational Study. Prog. Transpl..

[17] Spaderna H., Mendell N.R., Zahn D., Wang Y., Kahn J., Smits J.M., Weidner G. (2010). Social isolation and depression predict 12-month outcomes in the “waiting for a new heart study”. J. Heart Lung Transpl..

[18] Spaderna H., Weidner G., Koch K.C., Kaczmarek I., Wagner F.M., Smits J.M., Waiting for a New Heart Study Group (2012). Medical and psychosocial predictors of mechanical circulatory support device implantation and competing outcomes in the Waiting for a New Heart Study. J. Heart Lung Transpl..

[19] Spaderna H., Vogele C., Barten M.J., Smits J.M.A., Bunyamin V., Weidner G. (2014). Physical activity and depression predict event-free survival in heart transplant candidates. Health Psychol..

[20] Spaderna H., Weidner G., Zahn D., Smits J.M.A. (2009). Psychological characteristics and social integration of patients with ischemic and non-ischemic heart failure newly listed for heart transplantation: The Waiting for a New Heart Study. Appl. Psychol. Health Well-Being.

[21] Haneya A., Haake N., Diez C., Puehler T., Cremer J., Schmid C., Hirt S.W. (2011). Impact of the eurotransplant high-urgency heart allocation system on the outcome of transplant candidates in Germany. Thorac. Cardiovasc. Surg..

[22] Herrmann-Lingen C., Buss U., Snaith R.P. (2005). HADS-D Hospital Anxiety and Depression Scale—Deutsche Version. Ein Fragebogen zur Erfassung von Angst und Depressivität in der Somatischen Medizin.

[23] Herrmann C. (1997). International experiences with the Hospital Axiety and Depression Scale—A review of validation data and clinical results. J. Psychosom. Res..

[24] Frasure-Smith N., Lespérance F., Gravel G., Masson A., Juneau M., Talajic M., Bourassa M.G. (2000). Social support, depression, and mortality during the first year after myocardial infarction. Circulation.

[25] Dlugosch G.E., Krieger W. (1995). Fragebogen zur Erfassung des Gesundheitsverhaltens (FEG) [Questionnaire for the Assessment of health Behavior].

[26] Covas M.I., Konstantinidou V., Fito M. (2009). Olive oil and cardiovascular health. J. Cardiovasc. Pharm..

[27] Spaderna H., Zahn D., Pretsch J., Connor S.L., Zittermann A., Schulze Schleithoff S., Bramstedt K.A., Smits J.M., Weidner G. (2013). Dietary habits are related to outcomes in patients with advanced heart failure awaiting heart transplantation. J. Card Fail..

[28] Stewart A.L., Mills K.M., King A.C., Haskell W.L., Gillies D., Ritter P.L. (2001). CHAMPS Physical Activity Questionnaire for Older Adults: Outcomes for interventions. Med. Sci. Sports Exerc..

[29] Spaderna H., Zahn D., Schulze Schleithoff S., Stadlbauer T., Rupprecht L., Smits J.M.A., Krohne H.W., Münzel T., Weidner G. (2010). Depression and disease severity as correlates of everyday physical activity in heart transplant candidates. Transpl. Int..

[30] Ainsworth B.E., Haskell W.L., Whitt M.C., Irwin M.L., Swartz A.M., Strath S.J., O’Brien W.L., Bassett D.R., Schmitz K.H., Emplaincourt P.O. (2000). Compendium of physical activities: An update of activity codes and MET intensities. Med. Sci. Sports Exerc..

[31] Aaronson K.D., Schwartz J.S., Chen T.-M., Wong K.-L., Goin J.E., Mancini D.M. (1997). Development and Prospective Validation of a Clinical Index to Predict Survival in Ambulatory Patients Referred for Cardiac Transplant Evaluation. Circulation.

[32] Sterne J.A., White I.R., Carlin J.B., Spratt M., Royston P., Kenward M.G., Wood A.M., Carpenter J.R. (2009). Multiple imputation for missing data in epidemiological and clinical research: Potential and pitfalls. BMJ.

[33] Kim H.T. (2007). Cumulative incidence in competing risks data and competing risks regression analysis. Clin. Cancer Res..

[34] Fine J.P., Gray R.J. (1999). A Proportional Hazards Model for the Subdistribution of a Competing Risk. J. Am. Stat. Assoc..

[35] Scrucca L., Santucci A., Aversa F. (2010). Regression modeling of competing risk using R: An in depth guide for clinicians. Bone Marrow Transpl..

[36] Weidner G., Spaderna H. (2012). The role of the Heart Failure Survival Score and psychosocial stress in predicting event-free survival in patients referred for heart transplantation. J. Heart Lung Transpl..

[37] Freedland K.E., Hesseler M.J., Carney R.M., Steinmeyer B.C., Skala J.A., Dávila-Román V.G., Rich M.W. (2016). Major Depression and Long-Term Survival of Patients With Heart Failure. Psychosom Med..

[38] Celano C.M., Villegas A.C., Albanese A.M., Gaggin H.K., Huffman J.C. (2018). Depression and Anxiety in Heart Failure: A Review. Harv. Rev. Psychiatry.

[39] Freedland K.E., Carney R.M., Rich M.W., Steinmeyer B.C., Rubin E.H. (2015). Cognitive Behavior Therapy for Depression and Self-Care in Heart Failure Patients: A Randomized Clinical Trial. JAMA Intern. Med..

[40] Khan M.S., Jones D.W., Butler J. (2020). Salt, No Salt, or Less Salt for Patients With Heart Failure?. Am. J. Med..

[41] Mahtani K.R., Heneghan C., Onakpoya I., Tierney S., Aronson J.K., Roberts N., Hobbs F.D.R., Nunan D. (2018). Reduced Salt Intake for Heart Failure: A Systematic Review. JAMA Intern. Med..

[42] Frediani J.K., Reilly C.M., Higgins M., Clark P.C., Gary R.A., Dunbar S.B. (2013). Quality and Adequacy of Dietary Intake in a Southern Urban Heart Failure Population. J. Cardiovasc. Nurs..

[43] Mozaffarian D., Micha R., Wallace S. (2010). Effects on Coronary Heart Disease of Increasing Polyunsaturated Fat in Place of Saturated Fat: A Systematic Review and Meta-Analysis of Randomized Controlled Trials. PLOS Med..

[44] Guasch-Ferré M., Babio N., Martínez-González M.A., Corella D., Ros E., Martín-Peláez S., Estruch R., Arós F., Gómez-Gracia E., Fiol M. (2015). Dietary fat intake and risk of cardiovascular disease and all-cause mortality in a population at high risk of cardiovascular disease. Am. J. Clin. Nutr..

[45] Hooper L., Martin N., Jimoh O.F., Kirk C., Foster E., Abdelhamid A.S. (2020). Reduction in saturated fat intake for cardiovascular disease. Cochrane Database Syst. Rev..

[46] Pellicori P., Kaur K., Clark A.L. (2015). Fluid Management in Patients with Chronic Heart Failure. Card Fail. Rev..

[47] Abu-Sawwa R., Dunbar S.B., Quyyumi A.A., Sattler E.L.P. (2019). Nutrition intervention in heart failure: Should consumption of the DASH eating pattern be recommended to improve outcomes?. Heart Fail. Rev..

[48] Levitan E.B., Lewis C.E., Tinker L.F., Eaton C.B., Ahmed A., Manson J.E., Snetselaar L.G., Martin L.W., Trevisan M., Howard B.V. (2013). Mediterranean and DASH diet scores and mortality in women with heart failure: The Women’s Health Initiative. Circ. Heart Fail..

[49] Tuttolomondo A., Di Raimondo D., Casuccio A., Velardo M., Salamone G., Cataldi M., Corpora F., Restivo V., Pecoraro R., Della Corte V. (2020). Mediterranean diet adherence and congestive heart failure: Relationship with clinical severity and ischemic pathogenesis. Nutrition.

[50] Martínez-González M.A., Gea A., Ruiz-Canela M. (2019). The Mediterranean Diet and Cardiovascular Health. Circ. Res..

[51] Patel K., Sui X., Zhang Y., Fonarow G.C., Aban I.B., Brown C.J., Bittner V., Kitzman D.W., Allman R.M., Banach M. (2013). Prevention of heart failure in older adults may require higher levels of physical activity than needed for other cardiovascular events. Int. J. Cardiol..

[52] Doukky R., Mangla A., Ibrahim Z., Poulin M.F., Avery E., Collado F.M., Kaplan J., Richardson D., Powell L.H. (2016). Impact of Physical Inactivity on Mortality in Patients With Heart Failure. Am. J. Cardiol..

[53] Hoffmann J.M., Hellwig S., Brandenburg V.M., Spaderna H. (2018). Measuring Fear of Physical Activity in Patients with Heart Failure. Int. J. Behav. Med..

[54] Spaderna H., Hoffman J.M., Hellwig S., Brandenburg V.M. (2020). Fear of Physical Activity, Anxiety, and Depression: Barriers to Physical Activity in Outpatients With Heart Failure?. Eur. J. Health Psychol..

[55] Ohiomoba R.O., Youmans Q.R., Akanyirige P.W., Ezema A.U., Anderson A.S., Bryant A., Jackson K., Mandieka E., Pham D.T., Raza Y. (2020). History of cigarette smoking and heart transplant outcomes. Int. J. Cardiol. Heart Vasc..

[56] Vorlat A., Even P., Devrieze Y., Buyens E., Vermeulen T., Rodrigus I., Heidbuchel H., Claeys M. (2021). The deleterious effects of smoking resumption after heart transplantation. Acta Cardiol..

[57] Aune D., Schlesinger S., Norat T., Riboli E. (2019). Tobacco smoking and the risk of heart failure: A systematic review and meta-analysis of prospective studies. Eur. J. Prev. Cardiol..

[58] Dew M.A., DiMartini A.F., Dobbels F., Grady K.L., Jowsey-Gregoire S.G., Kaan A., Kendall K., Young Q.R., Abbey S.E., Butt Z. (2018). The 2018 ISHLT/APM/AST/ICCAC/STSW Recommendations for the Psychosocial Evaluation of Adult Cardiothoracic Transplant Candidates and Candidates for Long-term Mechanical Circulatory Support. Psychosomatics.

